# P-1473. Estimated Relative Effectiveness and Public Health Impact of Cell-Based Versus Egg-Based Influenza Vaccines During the 2023–2024 Season in the United States

**DOI:** 10.1093/ofid/ofaf695.1659

**Published:** 2026-01-11

**Authors:** Alicia N Stein, Anusorn Thanataveerat, Kimberly McDermott, Alex Dean, Stephanie Wall, Cory Pack, Sheena G Sullivan, Mahrukh Imran, Ian McGovern, Mendel Haag

**Affiliations:** CSL Seqirus, Melbourne, Victoria, Australia; Veradigm, New York, New York; Veradigm, New York, New York; Veradigm, New York, New York; Veradigm, New York, New York; Veradigm, New York, New York; Monash University, Melbourne, Victoria, Australia; CSL Seqirus, Melbourne, Victoria, Australia; CSL Seqirus, Melbourne, Victoria, Australia; CSL Seqirus, Melbourne, Victoria, Australia

## Abstract

**Background:**

Egg-adaptive mutations can alter the antigenicity of egg-based influenza vaccines, contributing to reduced vaccine effectiveness. Use of cell-based (QIVc) compared to egg-based (QIVe) quadrivalent influenza vaccines can improve effectiveness against test-confirmed influenza, as demonstrated during the United States (US) 2017–18 to 2019–20 and 2022–23 influenza seasons. Here we estimate the relative vaccine effectiveness (rVE) and potential public health impact of QIVc versus QIVe during the 2023–24 season.

**Figure:**
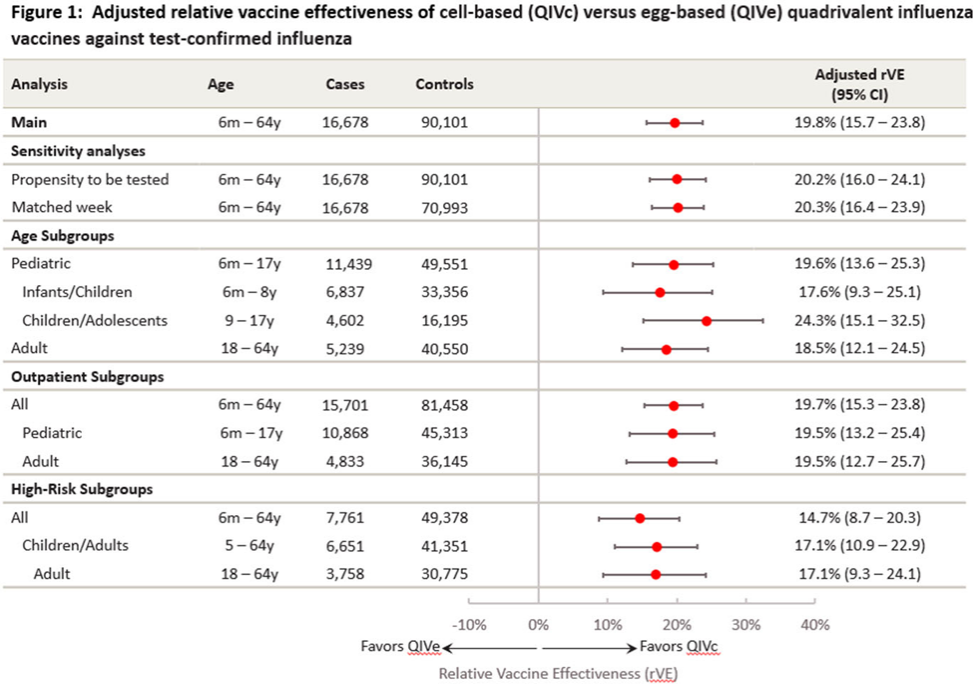

**Figure:**
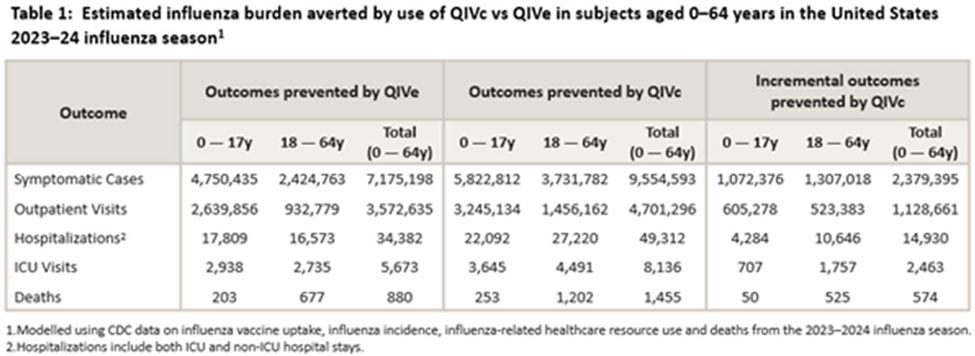

**Methods:**

rVE was estimated using linked data from electronic health records, medical and pharmacy claims and laboratory tests in the US. A retrospective test-negative design was applied among individuals aged 6 months–64 years vaccinated with QIVc or QIVe in the 2023–24 season and tested for influenza within 7 days of an acute respiratory or febrile illness (ARFI). rVE was estimated using doubly robust logistic regression in the full population, pediatric and adult subpopulations, individuals with high-risk conditions and those tested in outpatient settings. Sensitivity analyses adjusted for propensity to be tested and matched on test week. The public health impact was estimated using a compartmental influenza burden averted model.

**Results:**

The final dataset comprised 106,779 vaccinated ARFI patients, including 2,119 (13%) test-positive cases and 14,750 (87%) test-negative controls in the QIVc group and 14,559 (16%) cases and 75,351 (84%) controls in the QIVe group. QIVc was significantly more effective than QIVe in preventing test-confirmed influenza with an estimated rVE of 19.8% (95% CI: 15.7–23.8%) in the full population. Consistent rVE results were observed for sensitivity analyses and all subpopulations (Figure 1). If all vaccinated individuals aged 6 months–64 years in the US received QIVc over QIVe, an additional estimated 2,379,395 symptomatic illnesses would have been prevented, with proportionate reductions in related complications (Table 1).

**Conclusion:**

This study demonstrates superior effectiveness of QIVc versus QIVe in preventing test-confirmed influenza in people aged 6 months–64 years and across pediatric, adult, high-risk and outpatient subgroups, during the US 2023-24 season.

**Disclosures:**

Alicia N. Stein, MBiostats, PhD, CSL Seqirus: Grant/Research Support|CSL Seqirus: Stocks/Bonds (Private Company) Anusorn Thanataveerat, DrPH, Seqirus: Grant/Research Support Kimberly McDermott, PhD, CSL Seqirus: Advisor/Consultant Alex Dean, MPH, CSL Seqirus: Advisor/Consultant Stephanie Wall, MPH, CSL Seqirus: Advisor/Consultant Cory Pack, BS, Seqirus: Contracted Research Support Sheena G. Sullivan, PhD, Astra-Zeneca: Advisor/Consultant|CSL Behring and CSL Seqirus: Advisor/Consultant|GSK: Advisor/Consultant|Moderna: Advisor/Consultant|Novavax: Advisor/Consultant|Pfizer: Advisor/Consultant|Sanofi: Advisor/Consultant Mahrukh Imran, MScPH, CSL Seqirus: Grant/Research Support|CSL Seqirus: Stocks/Bonds (Private Company) Ian McGovern, MPH, CSL Seqirus: Grant/Research Support|CSL Seqirus: Stocks/Bonds (Private Company) Mendel Haag, PhD, PharmD, CSL Seqirus: Grant/Research Support|CSL Seqirus: Stocks/Bonds (Private Company)

